# Role of Circulating Immune Complexes in the Pathogenesis of Canine Leishmaniasis: New Players in Vaccine Development

**DOI:** 10.3390/microorganisms9040712

**Published:** 2021-03-30

**Authors:** Cristina Cacheiro-Llaguno, Nuria Parody, Marta R. Escutia, Jerónimo Carnés

**Affiliations:** R&D Unit Allergy & Immunology, LETI Pharma, S.L.U., Tres Cantos, 28760 Madrid, Spain; ccacheiro@leti.com (C.C.-L.); nparody@leti.com (N.P.); mroman@leti.com (M.R.E.)

**Keywords:** canine leishmaniasis, circulating immune complexes, biomarkers, diagnostic, *Leishmania infantum*, vaccines

## Abstract

During canine visceral leishmaniasis (CanL), due to *Leishmania infantum* (*L. infantum*), uncontrolled infection leads to a strong humoral immune response. As a consequence of the production of high antibody levels and the prolonged presence of parasite antigens, circulating immune complexes (CIC) are formed, which can be deposited in certain organs and tissues, inducing vasculitis, uveitis, dermatitis and especially glomerulonephritis and renal failure. A method to detect CIC and quantify their levels in serum samples from dogs infected with *L. infantum* has been recently described. It allowed demonstration of a correlation between CIC levels and disease severity. Thus, CIC measurement may be useful for diagnosis, assessment of disease progression and monitoring response to treatment. This is an interesting finding, considering that there remains an urgent need for identification of novel biomarkers to achieve a correct diagnosis and for optimal disease staging of dogs suffering from *Leishmania* infection. The objective of the present review is to shed light on the role of CIC in CanL, as well as to highlight their potential use not only as diagnostic and prognostic biomarkers but also as a valuable tool in vaccine development and new immunotherapy strategies to prevent or control disease outcome.

## 1. Introduction

Leishmaniases are a group of parasitic diseases caused by different species of the *Leishmania* parasite. Among them, canine visceral leishmaniasis (CanL), caused by *Leishmania infantum* (*L. infantum*), is a global zoonotic disease that is potentially fatal for dogs and, due to its potential transmission, to humans. The role of dogs as the main vertebrate reservoir is well-established, making visceral leishmaniasis (VL) a prime example of the importance of embracing a “One Health” approach for efficient surveillance and control of canine and human disease [1,2,3,4].

In CanL, one of the main determinants for the establishment of the infection is the host’s immune system’s ability to control the parasite. Two well-recognized types of host immune response are induced as a consequence of infection. On one hand, resistant dogs develop a robust Th1 immune response, resulting in the production of proinflammatory cytokines, such as IFN-γ and TNF-α, which limit the infection and associated inflammation by increasing the leishmanicidal activity of macrophages. In contrast, susceptible dogs develop a systemic immune response dominated by Th2 cells, regulatory T-cells (Tregs) and regulatory B cells [5,6]. The cytokines released by Th2 cells, which include interleukins IL-4 and IL-13, and the activity of Tregs and regulatory B cells via IL-10, downregulates the protective Th1 immune response, promoting “inappropriate” humoral immune responses [7,8,9,10,11,12,13]. The uncontrolled concentration of antibodies and the presence of *Leishmania* antigens induce the formation of circulating immune complexes (CIC) [14], composed of aggregated *Leishmania* proteins, anti-*Leishmania* IgG and IgM and, to a lesser extent, complement system fractions [15]. Macrophages activated by these immune complexes inhibit IL-12 biosynthesis, and, therefore, IFN-γ production, and secrete high levels of IL-10 [16], thus impeding the establishment of cell-mediated immunity and reducing the macrophage’s ability to kill the parasite [14]. Macrophages lose their ability to eliminate immune complexes, resulting in a deposition of CIC in the vascular walls of specific organs that leads to inflammation and tissue injury [14,17]. This deposition is responsible for some of the clinical manifestations of CanL [18], including glomerulonephritis, considered to be the most severe complication of CanL. It has variable clinical presentations, depending on the disease stage [12,14,19] and is the most frequent cause of renal failure and death.

CanL has a broad range of nonspecific clinical manifestations, ranging between subclinical, chronic and severe, sometimes reaching an acute stage that may kill the animal [20]. Therefore, the management of CanL is complex, and it is important to establish a standardized clinical staging system [12,21]. Veterinarians should use information from multiple sources, such as clinical history; examination findings; clinicopathological abnormalities; molecular tests to detect the parasite, such as the Polymerase Chain Reaction (PCR); and serological tests to evaluate the host immune response, such as the immunofluorescence antibody test (IFAT) or the Enzyme-Linked ImmunoSorbent Assay (ELISA). This evaluation is necessary to characterize the severity of the disease and determine the clinical stage, enabling the selection of an adequate treatment or to predict progression toward more serious and irreversible stages [19,20].

Although there are a wide variety of diagnostic techniques for CanL, none of them offers 100% sensitivity or specificity [22,23], and new diagnostic tools are needed to improve detection, especially in asymptomatic dogs. In this sense, Parody et al. recently described a method to isolate CIC and quantify their levels in serum samples obtained from dogs infected with *L. infantum* [15]. Furthermore, this study demonstrated a clear correlation between CIC levels and pathologic stage in animals infected with *L. infantum*. This fact has also been corroborated by other authors [14], suggesting that CIC analysis may have prognostic value. This is significant, considering that prediction of CanL clinical outcomes has been challenging to date, relying on the severity of clinicopathological abnormalities, in particular, those reflecting renal function, and the response to treatment, as prognostic indicators [12,18]. Interestingly, it has recently been published that vaccination with LetiFend^®^ reduces CIC levels, which may be related with the mechanism of control of *L. infantum* infection in dogs, although the mechanism of action has not yet been defined [18].

Therefore, CIC have been revealed as biomarkers with potential diagnostic and prognostic applications, and their measurement may allow not only improved disease staging but also improvements to diagnosis of clinically healthy dogs infected by *L. infantum*, in order to monitor disease progression and/or response to treatments.

The goal of this review is to summarize what constitutes the state-of-the-art regarding the role of CIC in CanL and to postulate how the analysis of these molecules can provide interesting biomarkers to gain more information about new players for diagnosis, prognosis and treatment of the disease.

## 2. Role of CIC in CanL

CanL is characterized by a large variety of clinical signs and clinicopathological alterations, the majority of which result from immune mediated mechanisms. Many of these alterations are attributed to CIC formation and deposition in specific tissues [24], causing vasculitis, polyarthritis, uveitis, meningitis and glomerulonephritis, which can cause proteinuria and may progress to renal failure and eventually death [25,26,27,28,29].

**Figure 1:**
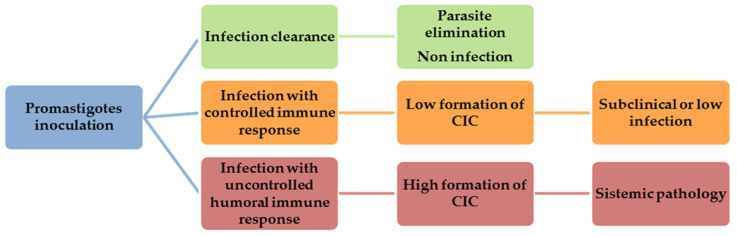
Canine visceral leishmaniasis (CanL) development after promastigotes inoculation.

The marked humoral response and ensuing CIC deposition in target organs of susceptible dogs constitute the mainstay of CanL pathogenesis, explaining the broad clinical spectrum observed [30,31]. The nature of the individual immune response determines whether the infection will be successfully controlled or whether the dogs will develop clinical signs due to the deposition of CIC (Figure 1).

CIC play an important role not only in leishmaniasis but also in many other infectious diseases [32,33]. Their presence has been reported in the serum of patients with a variety of pathologies caused by viral, bacterial, protozoal and helminthic agents. Immune complex-mediated pathologies can cause damage in different tissues and organs through complement activation, with or without local deposition, and can also modulate humoral and cellular immune responses through binding to surface receptors of lymphocytes and phagocytes [34].

Previous studies have highlighted the importance of CIC in the pathogenesis of a variety of systemic disorders, such as autoimmune diseases [35,36,37,38], allergic diseases [39,40], cancer [41,42] and infectious diseases [43,44,45]. In the latter, CIC have a limited capability of penetrating the basement membrane. However, in leishmaniasis, intense parasitic destruction liberates antigens, and consequently, CIC are formed, which circulate and easily penetrate the membrane, increasing subepithelial deposits [46,47]. In addition to CanL, a key role of CIC has been suggested in the pathogenesis of other canine vector- borne diseases, such as *Ehrlichia canis* and *Dirofilaria immitis* infections [48,49].

The role of CIC in the infection caused by *Leishmania* parasites has been studied in animal models and also in humans [16,50,51]. CIC were found in 30% of sera from human patients with VL. They were also detected in sera from patients with cutaneous leishmaniasis and persist in sera from clinically cured subjects [52]. These studies suggest that disease complications may be partly accounted for by CIC deposition-related pathology, particularly nephritis [27,29,53,54,55]. In this process, it has been shown that CIC size, IgG subclasses and glycosylation of IgG are relevant [56,57]. Of all the mechanisms that lead to development of renal pathology, those of an immunologic nature are the most important and involve many processes that have in common the deposition of immune complexes on glomerular walls and/or mesangial matrix [58].

CIC have been detected in sera from *Leishmania*-infected dogs [14,15,18,54,59], and the pathogenesis of renal lesions has been mainly attributed to CIC deposition within capillary beds of the glomerular tuft and subsequent glomerular injury [29,60,61,62,63,64,65,66,67,68,69]. In fact, CIC related renal pathology plays a pivotal role in prognosis, and it has been adopted as a major criterion for clinical staging of the disease in dogs [19,20,70,71,72].

## 3. Relationship between CIC Levels and Severity of CanL

In CanL, sick dogs usually produce high levels of *Leishmania*-specific immunoglobulins, which may give rise to CIC [29,62,63,64,73], whose defective clearance induces vasculitis, and their accumulation in specific organs leads to subsequent glomerular injury [27,29,54,55]. In this sense, CIC-related renal pathology in VL has been reported in animal models and also in humans [16,50,51].

Prior studies had suggested a relationship between CIC concentration and disease progression [16,62] but were not able to establish a direct correlation between clinical signs in CanL and CIC concentration.

More recently, a protocol to isolate and quantify the serum CIC levels in dogs naturally infected by *L. infantum* was developed [15]. The process provides levels of PEG-precipitated CIC, specific to *Leishmania* infection, based on a colorimetric assay and expressed as optical density, providing a useful tool for measuring the concentration in serum samples from animals in different sick stages (SS). A total of 60 dogs, classified according to the proposed LeishVet classification criteria (healthy, noninfected [*n* = 13]; infected, asymptomatic [*n* = 12]; SS I [*n* = 9]; SS II [*n* = 17]; SS III [*n* = 8]; and SS IV [*n* = 1]), were included in the study, and CIC levels were measured in order to correlate their levels and disease progression. This approach enabled a statistically significant correlation between the levels of CIC and pathologic stage in animals infected with *L. infantum* (see Table 1).

Moreover, this study revealed a significant (*p* < 0.001) positive correlation between IFAT titers, (the “gold standard” test in CanL) and CIC levels in animals from groups SS II and SS III/IV. In the same study, nanoparticle tracking analysis (NTA) technology, a powerful technique to analyze protein aggregates, was used to study CIC size in PEG-precipitated serum samples [74]. The data showed that dogs with more severe clinical signs (groups SS II and SS III/IV) present larger-sized CIC (ranging from 100 to 400 nm) during worsening of the disease. Further, a positive correlation between the size of analyzed CIC and the severity of the disease was demonstrated, supporting a pathogenic role of CIC in CanL, as previously published in other pathologies [75,76,77]. These data agree with previously published data, indicating that CIC size is also related to the deposition site, which plays a relevant role in the glomerular pattern of injury in *Leishmania*-infected dogs [27,29,57,61,63,66].

On the other hand, Gizzarelli et al. reported the nonspecific assessment of CIC levels by ELISA in sera from dogs naturally or experimentally infected with *Leishmania*. The results indicated a significant positive correlation between CIC values and IFAT titers, with low CIC concentration in clinically healthy infected dogs without or with low antibody response against *Leishmania*. Sera from dogs naturally infected showed significantly higher CIC levels than those from dogs experimentally infected. The main limitation of this approach is that it provides a nonspecific assessment of CIC independently from the pathogen that caused them, which is a pitfall in cases of co-infectious states [14].

## 4. CIC as Biomarkers for Measuring CanL Progression

Over the course of its lifetime, a *Leishmania*-infected dog may fit into several different categories, depending on its immune response and/or the parasite challenge or exposure [78]. The complexity of parasite–host interactions has revealed that a single biomarker cannot be used alone for CanL diagnosis or prognosis, and there is a great unmet need for new biomarkers, particularly serological ones, that involve noninvasive sampling [22,79].

In this sense, recent studies have analyzed biomarkers, incorporating them into vaccine immunogenicity and protection evaluations. Different biomarkers, such as cytokines patterns, tissue parasitism, leukocytes immunophenotyping or in vitro coculture systems using T-cells and macrophages infected with *L. infantum*, have been described in dog models in order to assess the immunogenicity and the protection elicited by vaccines against CanL in clinical trials [80].

Nevertheless, new biomarkers for the confirmation of *Leishmania* infection and useful to monitor disease progression during the treatment not only in sick animals but also in subclinical infected dogs would be a valuable tool to assist veterinarians in the control of CanL.

Although different published studies have suggested that CIC concentration is related to CanL progression [16,62], until now, this hypothesis had not been proven. Recently, different methods to isolate and quantify CIC in experimentally [18] and naturally infected dogs [14,15] have been described. These studies have established a positive correlation between CIC levels and disease stage, suggesting a potential diagnostic and prognostic value of CIC measurement in CanL. However, these results need a clinical validation for an accurate comparison of in vitro results with clinical characteristics of infected dogs. 

Recent advances have been made in biomarkers related to *Leishmania* pathogenesis in different organs and tissues. However, all of them involve invasive sampling and most cannot be used in a laboratory setting [22]. For this reason, a noninvasive method to isolate CIC and quantify their levels in serum samples [14,15] would have several advantages, being a valuable tool to monitor disease progression (see Table 2). 

Furthermore, the measurement of CIC as biomarkers for disease progression may provide interesting information regarding the ability of vaccines or immunotherapeutic treatments to control the disease. This is a relevant point, bearing in mind that vaccination is recognized as one of the most appropriate tools for prevention and remains the most promising approach to reduce the number of CanL cases and, therefore, the incidence of leishmaniasis in humans [8,81,82].

CIC may become relevant biomarkers in prophylaxis, considering that previous studies have postulated that immunization with internal parasite antigens [83,84,85,86,87] would stimulate a powerful immune response capable of reducing the formation of CIC. This would be in agreement with the hypothesis proposed by Chang et al., according to whom, in addition to surface and secretory products of the parasite, the origin of *Leishmania* virulence may also involve conserved intracellular proteins, referred to as “pathoantigens” [88]. In this context, it is most likely that immunization with internal antigens would elicit a strong and quick immune response, generating specific antibodies that clear parasite intracellular antigens and, therefore, significantly reducing the amount of CIC bound to pathology.

Although a clinical validation of CIC measurement is needed to correlate in vitro observations with clinical severity, these results suggest that CIC monitoring could represent an important tool in the management and prevention of CanL, not only because of their potential as diagnostic and prognostic biomarkers, but also because it may improve the development of vaccines or strategies for immunotherapy.

## 5. Conclusions

Different studies have highlighted the role of CIC in the pathogenesis of several diseases, including leishmaniasis. It has been shown that when CIC are not cleared by phagocytosis, they remained in blood circulation and can deposit within specific organs, resulting in inflammation and tissue injury. In CanL, it has been demonstrated that CIC concentration is clearly related to disease progression, playing a pivotal role in the most serious clinical manifestations of CanL, such as glomerulonephritis. These findings open new and exciting perspectives for research on the role of CIC in the pathology of CanL and their significance for further progress on vaccine development and new therapies.

## Figures and Tables

**Table 1:** CanL classification reflecting serological status, CIC levels, clinical signs and prognosis for each stage (based on LeishVet group guidelines for the practical management of CanL [20], and on published data regarding the relationship between circulating immune complexes (CIC) levels and the progression of CanL in naturally infected dogs [15]).

Clinical Stages	Serology	CIC	Clinical Signs	Prognosis
Noninfected	Negative	Negative	Without clinical signs	Good
Infected asymptomatic	Negative	Negative	Without clinical signs	Good
Stage I (Mild disease)	Negative or low positive antibody levels	Low levels	Mild clinical signs such as peripheral lymphadenomegaly or papular dermatitis	Good-to-guarded
Stage II (Moderate disease)	Low to high positive antibody levels	Low to medium levels	Apart from the signs listed in stage I, may present: diffuse or symmetrical cutaneous lesions such as exfoliative dermatitis/onychogryphosis, ulcerations (planum nasale, footpads, bony prominences, mucocutaneous junctions), anorexia, weight loss, fever and epistaxis	Guarded-to-good
Stage III (Severe disease)	Medium to high positive antibody levels	Medium to high levels	Apart from the signs listed in stages I and II, may present signs related to CIC deposition: vasculitis, arthritis, uveitis and glomerulonephritis.	Guarded-to-poor
Stage IV (Very severe disease)	Medium to high positive antibody levels	Medium to high levels	Clinical signs listed in stage III and pulmonary thromboembolism or nephrotic syndrome and end-stage renal disease	Poor

**Table 2:** Advantages of CIC analysis in CanL.

Advantages of CIC Analysis in CanL
✓ Noninvasive sampling
✓ High CIC levels in the presence of compatible clinical signs and/or clinicopathological abnormalities are conclusive of clinical leishmaniasis
✓ Biomarker associated to pathology
✓ Prognostic value
✓ Useful tool to monitor the treatment and vaccine efficacy
